# Visuomotor Integration for Coupled Hand Movements in Healthy Subjects and Patients With Stroke

**DOI:** 10.3389/fbioe.2020.00591

**Published:** 2020-06-30

**Authors:** Marco Iosa, Sheida Ghanbari Ghooshchy, Giovanni Morone, Pierluigi Zoccolotti, Simone Franceschilli, Fabiano Bini, Franco Marinozzi, Ugo Della Croce, Stefano Paolucci, Andrea Cereatti

**Affiliations:** ^1^IRCCS Fondazione Santa Lucia, Rome, Italy; ^2^Department of Psychology, Sapienza University of Rome, Rome, Italy; ^3^Department of Mechanical and Aerospace Engineering, Sapienza University of Rome, Rome, Italy; ^4^Department of Biomedical Sciences, University of Sassari, Sassari, Italy

**Keywords:** motor control, biomechanics, sensorimotor integration, stroke, rehabilitation

## Abstract

Many studies have investigated the bilateral upper limb coordination during movements under different motor and visual conditions. Bilateral training has also been proposed as an effective rehabilitative protocol for patients with stroke. However, the factors influencing in-phase vs. anti-phase coupling have not yet been fully explored. In this study, we used a motion capture device based on two infrared distance sensors to assess whether the up and down oscillation of the less functional hand (the non-dominant one in healthy younger and older subjects and the paretic one in patients with stroke) could be influenced by in-phase or anti-phase coupling of the more functional hand and by visual feedback. Similar patterns were found between single hand movements and in-phase coupled movements, whereas anti-phase coupled movements were less ample, less sinusoidal, but more frequent. These features were particularly evident for patients with stroke who showed a reduced waveform similarity of bilateral movements in all conditions but especially for anti-phase movements under visual control. These results indicate that visuomotor integration in patients with stroke could be less effective than in healthy subjects, probably because of the attentional overload required when moving the two limbs in an alternating fashion.

## Introduction

In the 1970s, Luria proposed the concept of “kinetic melody” to describe the orchestration of single motor impulses in a harmonic and fluid unique movement (Luria, 1973). This concept can easily be enlarged to bilateral movements, when the movement of a limb should be synchronized, in phase, or in anti-phase with the contralateral limb. Different neural processes seem to govern these two types of synchronization, with the movements of both limbs elicited by a common neural generator in bilateral in-phase movements and limbs controlled more independently during anti-phase movements (Shih et al., 2019). This could explain the higher variability of movements observed during anti-phase compared to in-phase movements (Shih et al., 2019). In-phase hand movements coupled with the contralateral hand can also be favored by the same biomechanical characteristics of the synchronous muscular patterns of the two upper extremities. Furthermore, kinesthetic afferences from one limb can influence the motor pathways to the other one (Baldissera et al., 1998, 2002; Cerri et al., 2003; Borroni et al., 2004).

Another system playing a key role is the visual system that contributes to control hand movements by comparing the intended with the actual position (Saunders and Knill, 2004). The sign and size of the detected position error would, in turn, determine the agonist vs. antagonist direction and amount of compensatory motor activation. By this position controller, each limb may overcome the mechanical contingencies by a continuous adjustment of the movement to the central motor command (Baldissera et al., 2006).

The visuomotor control in bilateral movements should occur in correspondence within the spatial domain (same amplitudes), the temporal domain (same frequencies), and the kinetic one (same forces), but it may also occur just in one or two of these domains and not in all of them. A study on repetitive bimanual circle-drawing task showed that somatosensory feedback was critical for spatial consistency but not for temporal coupling (Spencer et al., 2005). Another study highlighted that the orchestration of simultaneous reaching movements is based on the control of isomorphic movements of two limbs obtained by one single motor program, in which the temporal aspects are specified by common parameters, while force is coded by other, segment-specific, parameters (Schmidt et al., 1979).

In addition, the age of subjects may influence the visuomotor integration: older adults use visual information less effectively than younger adults to reduce force control error in bimanual pinch tasks (Critchley et al., 2014).

To couple the movements of both hands is even more complex when one is impaired, such as in patients with hemiparesis due to stroke; indeed, in these cases, the synchronization should take into account the functional asymmetry due to hemiparesis. Interestingly, in these patients, coupled bimanual training proved as a more effective intervention than unilateral training of the impaired hand (Rose and Winstein, 2004; Hung et al., 2019). Other studies reported a positive training effect of the not-affected hand on the affected one, thanks to the so-called “bilateral transfer” effect, *id est* the ability to transfer the skill acquired with a hand to the other one (Ausenda and Carnovali, 2011; Iosa et al., 2013).

The aim of the present study was to investigate how visual feedback (present vs. absent) and motor condition (in-phase vs. anti-phase) may influence the oscillatory movements of the less functional upper limb in healthy subjects (non-dominant limb) and in patients with stroke (affected limb). According to previous studies on visuomotor control (Saunders and Knill, 2004) and bilateral control (Ausenda and Carnovali, 2011; Iosa et al., 2013), our hypothesis was that in-phase condition performed under visual control could be helpful for patients to perform the task more similarly to how healthy subjects do it.

## Materials and Methods

### Participants

Seventy-six participants (40 females: 53%) were enrolled and divided into three groups: a young adult group (YG) of 48 healthy subjects (YG, mean age: 27 ± 5 years, providing reference physiological values), a patients' group (PG) of 10 subjects with stroke (mean age: 63 ± 11 years, time from stroke: 105 ± 82 days), and an adult group (AG) of 18 healthy subjects age-matched (*p* = 0.278) with patients (mean age: 58 ± 11 years). Inclusion criteria for healthy subjects were age (18–39 for YG and 40–80 for AG), absence of any motor or cognitive pathology, and normal, or corrected to normal with glasses vision. The inclusion criteria for patients were established clinical diagnosis of stroke confirmed by neuroimaging, age between 40 and 80 years of age, time from acute event lower than 9 months, and presence of residual movements of the paretic hand allowing performing the task. According to these criteria, we enrolled only patients with a Modified Ashworth Spasticity Scale score equal to 1, 1+, or 2. Exclusion criteria were comorbidities such as parkinsonisms or orthopedic pathologies of the upper limb, moderate to severe cognitive deficits (highlighted by a Mini-Mental State test score <24), and presence of unilateral spatial neglect.

### Experimental Task

**Figure 1:**
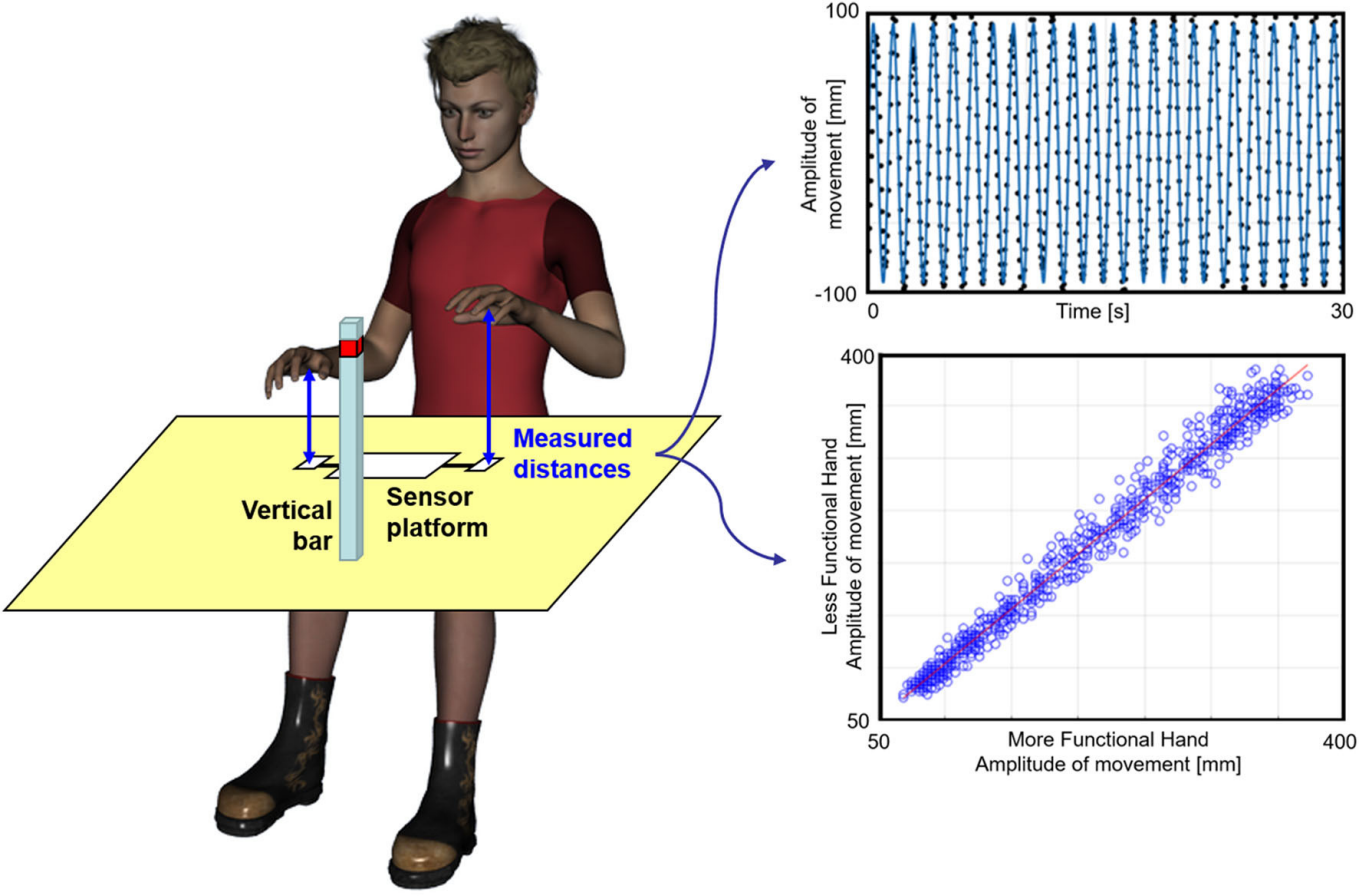
Experimental set-up. Left: a subject during the experiment. Top right: sinusoidal fit (blue line) computed for the distance between the hand and the sensor (black dots), normalized with respect to its mean value during an anti-phase movement performed under visual control by a young subject; bottom: application of the Linear Fit Method. The distance between less functional hand and sensor is plotted vs. the distance between the more functional hand and the other sensor (blue empty dots) for an in-phase coupled movement trial. The red line represents the linear regression fit from which the angular coefficient, the offset, and the coefficient of determination are extracted.

The subject sat in front of a table on which the sensor platform was placed (Figure 1). He/she was asked to keep the trunk maintaining it firm on the back of the chair. The subject was asked to oscillate his/her hand by moving it up and down along a vertical imaginary axis over the distance sensor, until a maximum height marked by a horizontal sign on a vertical bar posed in front of him/her (Figure 1). Subjects were asked to not overcome that sign on the bar, keeping them free to select the preferred amplitude of their movements within this limit. The movement had to be performed keeping the hand in prone position, with the palms downward, leaving the subject free to comfortably move the upper limb at the self-selected pace. The subject was required to perform the task in three different conditions: (1) movement performed by the less functional hand alone, (2) coupled in-phase movement, or (3) movement in anti-phase with the other hand. Two visual conditions were also tested: (1) open eyes and (2) closed eyes. Hence, six different conditions were tested, presented in a random sequence. The less functional hand was the paretic one for patients and the non-dominant hand for healthy subjects, as established by means of the Edinburgh Handedness Inventory (Oldfield, 1971).

### Instrumented Movement Analysis

To avoid applying sensors on the hand, as they could alter the ecological performance of subjects, we used a platform for range-distance measurements, the accuracy of which has already been validated in human movement analysis (Bertuletti et al., 2017, 2019). The Distance-MultiSensing platform integrates a magnetic and inertial measurement unit with a couple of Infrared Time-of-Flight (IR-ToF) proximity sensors. This latter technology provides an estimate of the distance between the sensor and the target (the hand) based on the time that an electromagnetic wave takes to travel that distance estimated by measuring the phase shift between the emitted and the reflected signals (Hansard et al., 2012). The two IR-ToF sensors were used to evaluate the distances of the two hands from the sensors with a sampling frequency of 25 Hz. The duration of each trial was 30 s, but the first and last parts of the trial were not analyzed for excluding accelerating and decelerating parts of the signal. For each trial, at least 15 s of steady state of the signal in the central part of the trial was analyzed. Then, the obtained data were filtered at 5 Hz using a fourth-order Butterworth filter. As shown in the top plot of Figure 1, the filtered signal was fitted with a sinusoidal curve, and the amplitude and frequency of this sinusoid were computed. The corresponding *R*^2^ was taken as an indicator of how much the movement was represented by its first sinusoidal harmonic (oscillation harmony). This method allowed to estimate the main amplitude of oscillations and preferred the peak-to-peak amplitude obtained by raw data that could be affected by only one wide oscillation, with results not representative of the whole task. For coupled movements, the linear fit method (LFM) was also applied for assessing the pattern similarity of the two hands as shown in the bottom plot of Figure 1 (Iosa et al., 2014, 2019). LFM is based on application of a linear fit to the set of data points obtained when a set of data (that of the less functional hand) is plotted on the y-axis vs. a reference set of data (the more functional hand) plotted on the x-axis. The former was the dependent variable and the latter the independent variable. The LFM minimizes, in a least square sense, the vertical distances between the points and the fitting line characterized by an angular coefficient representing the ratio among the amplitude of the two patterns. The intercept represents the eventual offset between the two patterns. The coefficient of determination represents the strength of the relationship between the two patterns. Altogether, these three parameters provide an in-depth assessment of waveform similarity.

### Statistical Analysis

Data were reported in terms of mean and standard deviation. A mixed analysis of variance (ANOVA) was used for assessing the effects of the group as between-subjects factor and of motor and visual conditions as within subjects' factors. Separate analyses of variance were carried out on amplitude, frequency, and *R*^2^-values. Effect sizes (ES) were evaluated as partial eta squared, representing the proportion of variance attributed to each effect. Where statistically significant, ANOVA was followed by *post-hoc* tests between groups. For the analyses of variance, the alpha level of significance was set at 5%, reduced in *post-hoc* tests by the application of Bonferroni correction.

## Results

**Figure 2:**
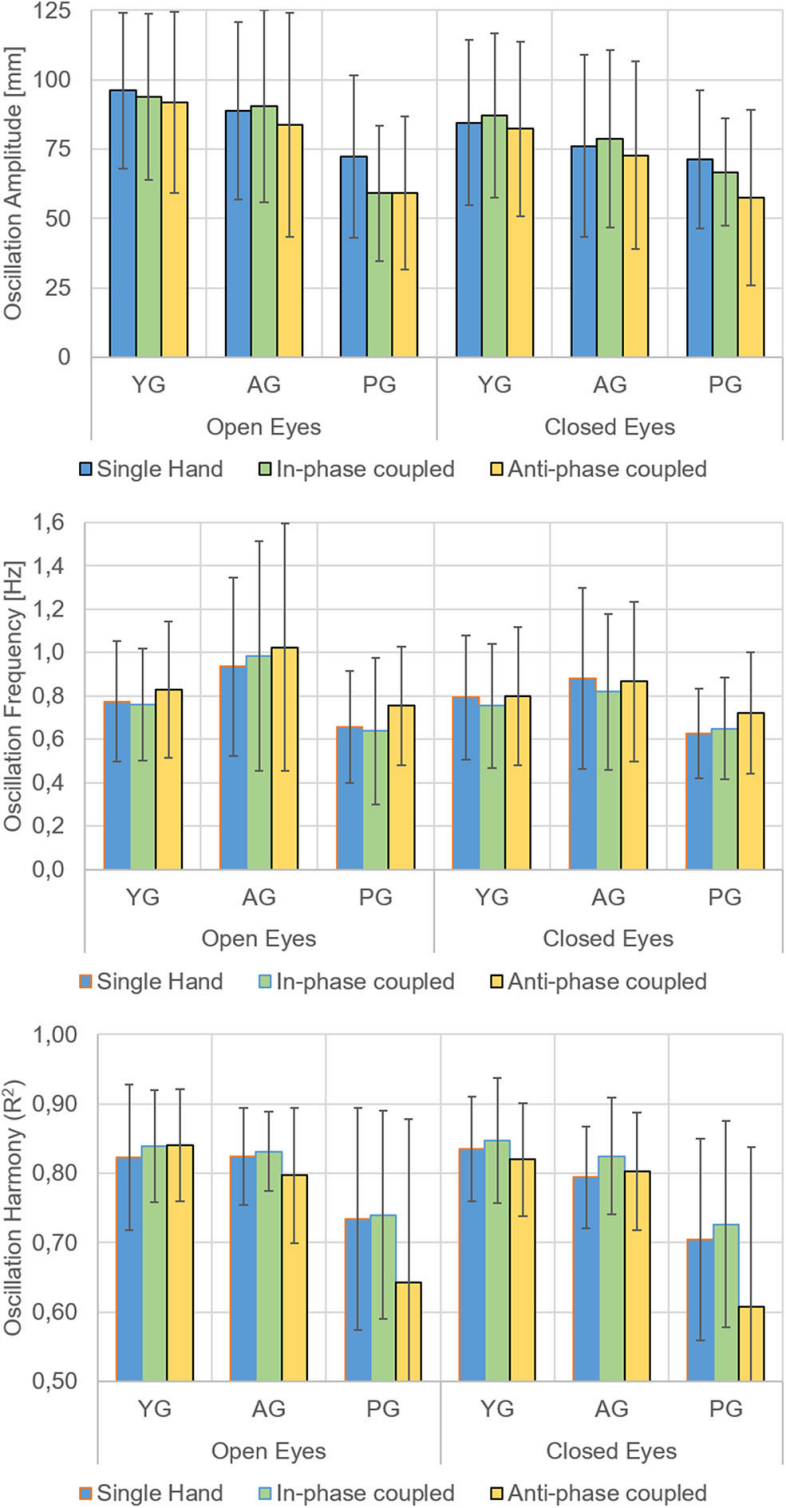
Main Results. Means and standard deviations of the oscillation amplitude (top), oscillation frequency (middle), and oscillation harmony (*R*^2^, bottom) of the less functional hand in the younger group (YG), in the age-matched adult group (AG), and in the patient group (PG). Open and closed eyes conditions and movement performed with a single hand (blue bars), with in-phase (green bars), and anti-phase (yellow bars) coupled movements with the more functional hand separately presented.

Relevant means for amplitude, frequency, and *R*^2^-values are presented in Figure 2 and analyses of variance on these values in Table 1. As for amplitude measures, the main effects of group, visual condition, and motor condition were all statistically significant; none of the interactions were significant. The ANOVA on the frequency of movements showed the main effects of the visual and motor conditions, but not of the group factor (*p* = 0.119); the interaction between group and visual conditions was reliable (*p* = 0.008). As for *R*^2^-values (indicating the regularity of sinusoidal oscillations), the ANOVA indicated the main effects of the motor condition and group factors as well as their interaction. The main effect of the visual condition was not significant (*p* = 0.283).

**Table 1:** Results of the analysis of variance: F-values, probability p, and effect size ES.

**ANOVA**	**Factors**		**Amplitude**	**Frequency**	***R*^**2**^**
Main effects	Vision	F	8.073	6.528	1.169
		p	**0.006**	**0.013**	0.283
		ES	0.100	0.082	0.016
	Motor	F	4.883	6.877	7.875
		p	**0.009**	**0.001**	**0.001**
		ES	0.063	0.086	0.097
	Group	F	3.526	2.191	20.858
		p	**0.035**	0.119	**<0.001**
		ES	0.088	0.057	0.364
Second-level interactions	Vision*Group	F	2.351	5.128	0.452
		p	0.102	**0.008**	0.638
		ES	0.061	0.123	0.012
	Motor*Group	F	1.135	0.584	3.011
		p	0.342	0.675	**0.020**
		ES	0.030	0.016	0.076
	Vision*Motor	F	0.994	1.409	0.207
		p	0.373	0.248	0.813
		ES	0.013	0.019	0.003
Third-level interaction	Vision*Motor*Group	F	0.301	0.931	0.697
		p	0.877	0.448	0.595
		ES	0.008	0.025	0.019

*Statistically significant (p < 0.05) are marked in bold*.

In the open-eye condition, the movements were wider, and there were more frequent and faster oscillations than in closed eyes condition. As for frequency, the effect of visual condition showed a significant interaction with group (*p* = 0.008).

With respect to movements performed with a single hand and with in-phase coupled hands, the anti-phase coupled movements were less wide and less regular, but more frequent. This last finding also depended upon the worst performance of patients in anti-phase coupled oscillations, as indicated by the significant motor^*^group (*p* = 0.020) interaction.

Patients had less wide and less regular oscillations of the affected hand with respect to the non-dominant hand of healthy subjects. The analysis of interactions also revealed that patients' performance in terms of frequency of oscillations depended on visual condition, whereas the regularity of oscillations was affected by motor condition.

The LFM analysis of synchronization (Table 2) of the two hands during coupled movements showed a reduction of waveform similarity in anti-phase coupled movements with respect to the in-phase ones. Patients were significantly different from YG in the regularity of oscillations (coefficient of determination) for all four visuomotor conditions (*p* < 0.004), as well as in terms of amplitude (*p* = 0.01) and offset (*p* = 0.003) in anti-phase coupled movements, but only under visual control. These last two parameters, in closed eyes condition, resulted more similar to AG and, especially, not significantly different from YG. Significant differences were also noted between PG and AG in the open eyes condition, for in-phase *R*^2^ (*p* = 0.013) and anti-phase offset (*p* = 0.014).

**Table 2:** Means ± standard deviations of the Linear Fit Method parameters for young group (YG), age-matched adult group (AG) and patients group (PG).

**LFM parameters**	**Visual condition**	**Motor condition**	**Theoretical value**	**YG**	**AG**	**PG**
Amplitude (angular coefficient)	Open eyes	In-phase	1	1.02 ± 0.06	1.05 ± 0.09	0.98 ± 0.19
		Anti-phase	−1	−0.99 ± 0.14	−1.05 ± 0.15	**−0.72** **±** **0.67**
	Closed eyes	In-phase	1	1.01 ± 0.07	1.01 ± 0.10	0.95 ± 0.27
		Anti-phase	−1	−0.91 ± 0.30	−0.87 ± 0.53	−0.85 ± 0.45
Offset (intercept)	Open eyes	In-phase	0	−2 ± 11	−6 ± 21	7 ± 24
		Anti-phase	–	356 ± 97	360 ± 98	**249** **±** 121*
	Closed eyes	In-phase	0	−1 ± 18	−4 ± 20	−2 ± 38
		Anti-phase	–	306 ± 99	278 ± 79	271 ± 116
Similarity (coefficient of determination)	Open eyes	In-phase	1	0.97 ± 0.01	0.96 ± 0.03	**0.88** **±** 0.14*
		Anti-phase	1	0.84 ± 0.11	0.83 ± 0.08	**0.70** **±** **0.21**
	Closed eyes	In-phase	1	0.95 ± 0.06	0.94 ± 0.05	**0.84** **±** **0.17**
		Anti-phase	1	0.78 ± 0.16	0.76 ± 0.13	**0.60** **±** **0.26**

*Values significantly different in AG and PG with respect to YG are marked in bold, whereas the stars show significant differences between PG and AG.*.

## Discussion

The aim of this study was to investigate how different visuomotor conditions may alter the movements of the non-dominant hand in healthy subjects and the affected hand in patients with stroke during up and down oscillations performed at a self-selected pace. For patients, in-phase condition resulted a motor facilitator as hypothesized, but the effect of visuomotor control depended on motor condition, in fact the performance surprisingly worsened in anti-phase movements. Sensory-motor integration is necessary during this task, and our results clearly showed an effect of visual feedback on the amplitude and frequency of movements and an effect of coupling movements with the other hand on amplitude, frequency and waveform of movements. Visual feedback had a positive effect increasing amplitude of movements and their frequency, but not changing the sinusoidal regularity of movements. As expected, this last parameter was highly reduced in patients, and just dependent on motor conditions: slightly increased in in-phase coupled oscillations in all groups, but drastically reduced in anti-phase oscillations only in patients. The only positive effect of the anti-phase oscillations was an increase in the frequency of movements, but this may be due to the reduced amplitude (the ampler is the movement, the longer is the time needed to perform it, and the smaller is the frequency). This could also be the reason why AG showed a slightly higher frequency than YG (Figure 1), despite the effect of group not being statistically significant.

The LFM analysis indicated that the values of young adults were close to the theoretical ones (Table 2), confirming the goodness of the applied method for assessing the synchronization of bilateral movements. In fact, YG had a slight deviation from theoretical values only for anti-phase coupled movements performed with closed eyes, confirming the efficacy of visual control in young adults for managing bimanual tasks (Critchley et al., 2014). Interestingly, the anti-phase coupled movements of patients were more similar between the two hands when performed with closed than with open eyes. This trend was opposite to that shown by younger and older healthy subjects, for whom visual control allowed obtaining values closed to those expected for perfectly synchronized movements.

A substantial overlap between the neural processes underlying bilateral and unilateral upper limb movements was found as to the possible basis of generalizing bilateral training to unilateral performance, but the extent of this generalization could depend on the breadth of visual experience obtained during bilateral training (Wang et al., 2013). In our study, visual control of two hands moving in anti-phase could be cognitively difficult for patients with stroke, leading to more synchronous anti-phase movements when eyes were closed. In fact, when bimanual movements are performed, the visuomotor integration might be easier if both limbs perform the same movement at the same time, with respect to anti-phase movements. An alternative hypothesis could be that our group of patients had some still undiagnosed visual or visuomotor integration deficits. However, this hypothesis seems to be less convincing as, in that case, also the in-phase coupled oscillations should had benefitted by closing the eyes, whereas this did not happen. Also, proprioception could play a key role. In our study, neither proprioception was tested nor the hand absolute position analyzed. Recently, position control related to proprioception was suggested to be preferred to amplitude kinesthetic control (Marini et al., 2018). Task-dependent asymmetries in proprioception were also reported between preferred and non-preferred limb (Goble and Brown, 2007). Vision could interfere with the proprioception in patients with stroke because the elaboration of both these signals could be altered, worsening the performance (Herter et al., 2019). This hypothesis is in line with the recent finding that integration does not always occur at the sensory information processing level, but it may happen at the motor execution level (Hayashi et al., 2020). An altered integration may also affect the motor memory for each sensory modality in bimanual and unimanual movements, especially in subjects post stroke. Despite the absence of a proprioception assessment was a limit of our study, in a further study, the Distance-MultiSensing platform, with properly developed protocols, could be used to assess proprioception in patients with stroke or other asymmetrical pathologies. A quantitative assessment of proprioception could also be integrated in a proprioceptive rehabilitative training for patients (Squeri et al., 2011).

Our results seem in line with those of the literature reporting that simultaneous and symmetrical recruitment of muscle groups occurring during in-phase movements may lead to more accurate and effortless movements than anti-phase ones, in which homologous muscle groups are activated in an alternating fashion (Wuyts et al., 1996; Serrien and Brown, 2002; Pollok et al., 2007). This effortlessness of in-phase movements is conceivably supported by our finding of the reduced synchronicity of the two hands in patients for anti-phase movements performed under visual control. This condition was probably inflated by higher attentional load required for controlling the two hands moving in opposition of phase. This interpretation is in line with previous findings showing that in-phase movements can be performed accurately without practice, while anti-phase movements often require training to be accurately performed (Lee et al., 1995). Furthermore, when subjects are required to increase the frequency of anti-phase movements, they unintentionally tend to change them into in-phase movements (Franz et al., 1991). It is also likely that in-phase movements are more easily managed by a common neural generator, probably guided by the dominant hemisphere, resulting in highly synchronous movements, while anti-phase movements are controlled by both hemispheres more independently leading to less synchronicity. Furthermore, we should take into account the possibility that different motor strategies were adopted in the different tasks of our study. During in-phase button press tasks, bimanual motor synergy was shown to be at the basis of bimanual isometric force control in healthy subjects (Jin et al., 2019). However, Zenzeri et al. (2014) showed that some motor behaviors cannot be easily explained in terms of a global optimization criterion but rather require the ability to switch between different sub-optimal mechanisms, switching among motor strategies under different conditions.

So, despite the evidence supporting bilateral arm training in patients with stroke (Cauraugh et al., 2010), further studies are required to investigate whether it is more effective to train patients using in-phase well-coordinated movements or the less effective anti-phase bilateral movements. Our study may contribute to this debate showing that visual control may reduce the synchronicity of anti-phase movements in patients, probably because of the higher cognitive load. In this scenario, further investigations on the potential effects of external visual or auditory cues in training patients with stroke during unilateral and bilateral hand movements would be needed.

Our study has some limitations, which suggest caution in data interpretation. First of all, the sample size was higher for YG than for PG: we needed to enroll a large sample of adults to obtain reference physiological data, but further studies should also use a larger sample of patients. A wider patient group together with a more detailed clinical assessment could provide more consistent data, clarifying the influence of motor and cognitive deficits, and allowing to test correlations between clinical features and task performances. Then, we also recorded the data of the more functional hand during coupling movements, but used them in LFM for evaluating the similarity with the less functional hand: for the sake of brevity, we did not report in this study the raw data of the functional hand. Finally, as stated above, the absence of proprioception assessment is another limit of our study.

In conclusion, our study confirmed previous results showing that in-phase coupled movements were more synchronized than anti-phase movements in all subjects. Furthermore, visual feedback actually worsened the performance of patients with stroke in anti-phase movements, conceivably because of the attentional overload required in this condition.

## Data Availability Statement

The datasets generated for this study are available on request to the corresponding author.

## Ethics Statement

The studies involving human participants were reviewed and approved by Independent Ethical Committee of Fondazione Santa Lucia. The patients/participants provided their written informed consent to participate in this study.

## Author Contributions

MI, GM, and SP conceived the study, interpreted the results, and drafted the manuscript. AC and UD designed the experimental setup. SG and SF enrolled patients and performed the data collection. PZ, FB, and FM aided in interpreting the results and contributed to the manuscript. All authors contributed to the article and approved the submitted version.

## Conflict of Interest

The authors declare that the research was conducted in the absence of any commercial or financial relationships that could be construed as a potential conflict of interest.
